# Perfusion Changes in Acute Stroke Treated with Theophylline as an Add-on to Thrombolysis: A Randomized Clinical Trial Subgroup Analysis

**DOI:** 10.1007/s00062-021-01029-x

**Published:** 2021-07-14

**Authors:** Boris Modrau, Anthony Winder, Niels Hjort, Martin Nygård Johansen, Grethe Andersen, Jens Fiehler, Henrik Vorum, Nils D. Forkert

**Affiliations:** 1 Department of Neurology, Aalborg University Hospital, Postbox 561, 9100 Aalborg, Denmark; 2 Departments of Radiology & Clinical Neurosciences, University of Calgary, Calgary, Canada; 3 Department of Neurology, Aarhus University Hospital, Aarhus, Denmark; 4 Unit of Clinical Biostatistics, Aalborg University Hospital, Aalborg, Denmark; 5 Department of Neurology and Clinical Medicine, Aarhus University Hospital, Aarhus University, Aarhus, Denmark; 6 Department of Diagnostic and Interventional Neuroradiology, University Medical Center Hamburg-Eppendorf, Hamburg, Germany; 7 Department of Ophthalmology, Aalborg University Hospital, Aalborg, Denmark

**Keywords:** Multi-parametric MRI, Infarct core, Penumbra, Neuroprotection, rtPa

## Abstract

**Purpose:**

Theophylline has been suggested to have a neuroprotective effect in ischemic stroke; however, results from animal stroke models and clinical trials in humans are controversial. The aim of this study was to assess the effect of theophylline on the cerebral perfusion with multiparametric magnetic resonance imaging (MRI).

**Methods:**

The relative cerebral blood flow (rCBF), relative cerebral blood volume (rCBV), and relative mean transit time (rMTT) in the infarct core, penumbra, and unaffected tissue were measured using multi-parametric MRI at baseline and 3‑h follow-up in patients treated with theophylline or placebo as an add-on to thrombolytic therapy.

**Results:**

No significant differences in mean rCBF, rCBV, and rMTT was found in the penumbra and unaffected tissue between the theophylline group and the control group between baseline and 3‑h follow-up. In the infarct core, mean rCBV increased on average by 0.05 in the theophylline group and decreased by 0.14 in the control group (*p* < 0.04). Mean rCBF and mean rMTT in the infarct core were similar between the two treatment groups.

**Conclusion:**

The results indicate that theophylline does not change the perfusion in potentially salvageable penumbral tissue but only affects the rCBV in the infarct core. In contrast to the penumbra, the infarct core is unlikely to be salvageable, which might explain why theophylline failed in clinical trials.

**Supplementary Information:**

The online version of this article (10.1007/s00062-021-01029-x) contains supplementary material, which is available to authorized users.

## Introduction

The neuroprotective effect of theophylline is controversial with conflicting results in animal ischemic stroke models and previous clinical trials in humans. Animal stroke models have demonstrated reduced ischemic brain damage, reduced perifocal edema, and reduced mortality by preconditioning or perconditioning with theophylline [1–3]; however, previous randomized clinical trials failed to demonstrate improved clinical outcome in humans [4, 5].1

Theophylline acts as a phosphodiesterase inhibitor and competitive adenosine receptor antagonist with well-documented effects in the respiratory system [6]; however, the vasoactive effect of theophylline on ischemic brain tissue is not fully understood. Inhibition of phosphodiesterase increases cyclic adenosine monophosphate that subsequently activates calcium-activated potassium channels and adenosine triphosphate-sensitive potassium channels, which causes vasodilatation [7]; however, inhibition of adenosine receptor causes cerebral vasoconstriction [8, 9]. This vasoconstriction was previously found in cortical tissue in animal stroke models and humans, but not in ischemic tissue [10, 11]. Regional cerebral blood flow (CBF) measured using the Xenon-133 arterial injection technique demonstrated that theophylline decreases regional CBF in areas of normal brain tissue and increases regional CBF in areas of stroke, a phenomenon described as inverse intracerebral steal [12].

In contrast to the previous studies, dynamic susceptibility contrast-enhanced magnet resonance imaging (MRI) allows not only the cerebral blood flow, cerebral blood volume (CBV), and mean transit time (MTT) [13] to be quantified but also to differentiate between the infarct core and the penumbra in patients with acute ischemic stroke [14]; however, this imaging technique has not been used to investigate potential effects of theophylline on the regional cerebral perfusion.

The aim of this study was to investigate the effect of theophylline on perfusion in the infarct core and penumbra in patients with acute ischemic stroke.

## Material and Methods

### Study Objective

This subgroup study is based on the theophylline in acute ischemic stroke trial, a proof-of-concept, randomized, double-blinded, placebo-controlled trial that assessed the neuroprotective effect of theophylline as an add-on to thrombolytic therapy (EudraCT number 2013-001989-42). The trial protocol was approved by the Danish Health and Medicines Authorities (ref. no. 2013050908) and the Regional Scientific Ethics Committee (ref.-no. N‑20130034) [15]. The main inclusion criteria were MRI verified moderate to severe stroke symptoms (National Institutes of Health Stroke Scale [NIHSS] ≥4), eligibility for thrombolytic therapy within 4.5 h of symptom onset and written informed consent. The trial was terminated early due to slow recruitment. The co-primary endpoints, early clinical improvement and infarct growth at 24 h follow-up, were without significant difference after correction for multiplicity [16].

This preplanned sub-group analysis included all patients with multiparametric MRI including perfusion-weighted imaging available at baseline and 3–4 h follow-up (in the following referred as 3‑h follow-up). This interval was chosen for follow-up imaging as theophylline is expected to be still active (half-life 3–9 h) at this time.

### Image Acquisition

Multiparametric MRI was performed with the same field strength (1.5 or 3.0 T) at baseline and at 3‑h follow-up including diffusion-weighted MRI (DWI), perfusion-weighted MRI (PWI) with intravenous gadolinium (0.1 mmol per kg body weight, 5 ml/s bolus injection), circle of Willis time-of-flight MR angiography (TOF MRA), and fluid-attenuated inversion recovery (FLAIR) MRI. The thrombolysis in myocardial infarction (TIMI) grading was used to grade arterial obstruction [17]. Large vessel occlusion was defined as TIMI 0–1 at baseline and conversion from TIMI 0–1 at baseline to 2–3 at 3‑h follow-up was defined as revascularization.

### Imaging Post-Processing

**Fig. 1:**
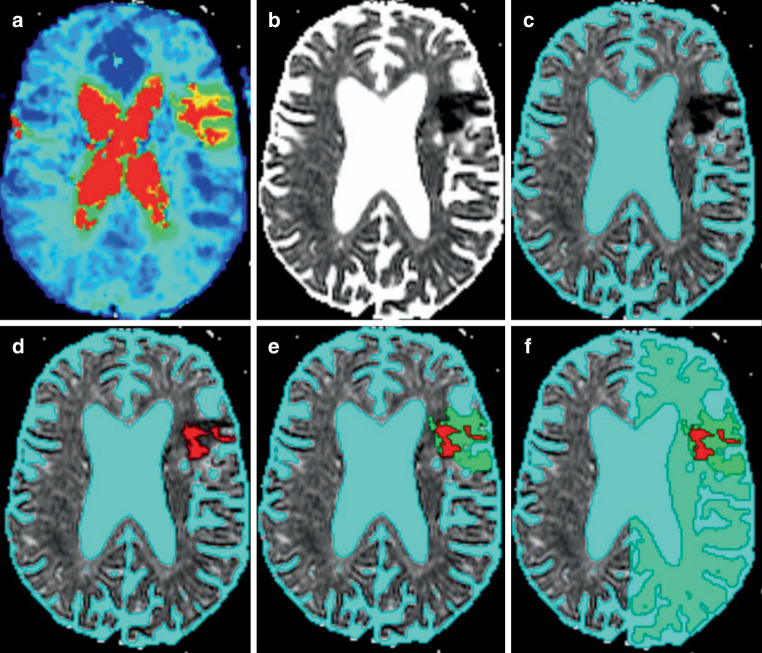
Axial section of the brain using multi-parametric MRI with semiautomatic imaging post-processing: **a** T-max map; **b** apparent diffussion (ADC) map; **c** ADC map with cerebrospinal fluid (CSF) segmentation; **d** infarct core region of interest (ROI) (*red*), **e** infarct core ROI (*red*) and penumbra ROI (*bright green*); **f** infarct core ROI (*red*), penumbra ROI (*bright green*), unaffected tissue ROI (*light green*)

Image analysis of the DWI and PWI MRI datasets was performed using the semi-automatic software tool AnToNIa [18]. In brief, the b0 and b1000 mm^2^/s DWI datasets were used to calculate an apparent diffusion (ADC) dataset. A semi-automatic volume growing approach with a rather conservative upper ADC threshold of 550 × 10^−6^ mm^2^/s, in accordance with previous stroke studies [19], was used to segment the infarct core region of interest (ROI). This threshold was used to ensure that no penumbra tissue is part of the infarct core. The PWI dataset was automatically motion corrected and the arterial input function was manually identified from the internal carotid artery and middle cerebral artery (M1 segment). After this, a block-circulant deconvolution-based perfusion analysis with a threshold of 0.15 was applied to calculate the CBF, CBV, MTT, and time to maximum (Tmax) of the residual function. After this, relative perfusion maps (rCBF, rCBV, rMTT, and rTmax) were computed using mean values from contralateral brain tissue, excluding cerebrospinal fluid, and registered to the ADC dataset. More precisely, subtraction was used for the temporal parameters (rMTT and rTmax), while division was used for calculation of the rCBV and rCBF maps. The rTmax map was then used to segment the hyperperfused tissue applying a semiautomatic volume growing approach with a lower Tmax threshold of 6 s [20]. The penumbra ROI was computed by subtracting the infarct core ROI from the hypoperfusion ROI (perfusion-diffusion mismatch). The unaffected tissue ROI was computed by subtracting infarct core and penumbra ROIs from the brain segmentation in the affected hemisphere. The mean rCBV, rCBF, and rMTT values were calculated for the infarct core ROI, penumbra ROI, and unaffected tissue ROI using the baseline relative perfusion parameter maps. The same baseline ROIs were applied to calculate the average perfusion parameters in the 3‑h follow-up PWI dataset registered to the acute ADC dataset (Fig. 1).

Thus, ADC, rCBF, rCBV, and rMTT values were available for the infarct core, penumbra, and the unaffected tissue at baseline and 3‑h follow-up.

### Clinical Assessment

Clinical improvement was described in several case series and non-randomized clinical trials immediately after administration of theophylline. For that reason, healthcare professionals blinded to the allocation of the study medication assessed the clinical improvement defined as improvement in the National Institute of Health Stroke Scale (NIHSS) score by ≥4 points from baseline to 3‑h follow-up. Furthermore, blood pressure and heart rate were measured at baseline and 3‑h follow-up to assess the positive inotropic and chronotropic effect of theophylline.

### Theophylline Concentration Assessment

A blood sample was taken to assess the blood serum concentration of theophylline at 3‑h follow-up.

### Statistical Analyses

Baseline characteristics were summarized by mean and standard deviation (SD) for continuous data and counts and percentages for categorical data. Continuous data were compared with a two-sided t‑test or Wilcoxon’s rank sum test and categorical data were compared using Fisher’s exact test.

A Wilcoxon’s rank sum test was used to compare baseline and follow-up rCBF, rCBV, and rMTT within the infarct core, penumbra, and unaffected tissue. A two-sided t‑test with equal variances was used to compare the differences in relative perfusion parameters from baseline to follow-up. All tests were applied as post hoc analyses as the preplanned substudy analysis was omitted due to slow recruitment and early termination of the trial. A two-sided alpha level of 0.05 without correction for multiple comparisons was applied for this explorative analysis. Stata/MP version 16.1 (Stata Corp LLC, College Station, TX, USA) was used for the statistical analysis.

Anonymized data that support the findings of this study are available from the corresponding author upon reasonable request.

## Results

### Study Population

An MRI at 3‑h follow-up was acquired and available in 24 out of 64 patients included in the theophylline as an add-on to thrombolytic therapy trial. Of the patients 13 were treated with theophylline and 11 patients were treated with placebo. Baseline patient characteristics, including baseline imaging parameters, process measures, and main follow-up characteristics were not significantly different between the two groups (Table 1). The change in heart rate from baseline to 3‑h follow-up was significantly different (*p* < 0.05) in the theophylline group (mean increase by 11 beats per minute, SD 26) compared to the control group (mean decrease by 9 beats per minute, SD 14). The change in systolic blood pressure (−12 mm Hg, SD 14 versus −8 mm Hg, SD 25; *p* = 0.62) and the diastolic blood pressure (−9 mm Hg, SD 8 versus −12 mm Hg, SD 12; *p* = 0.51) from baseline to 3‑h follow-up was similar in the theophylline group and the control group. Clinical improvement at 3‑h follow-up was present in 1 (8%) patient in the theophylline group and 2 patients (18%) in the control group (*p* = 1.0). Large vessel occlusion (LVO) at baseline was identified by TOF MRA in 11 patients. Recanalization was found in 7 of 7 patients with LVO in the theophylline group and in 2 of 4 in the control group (*p* = 0.11). The mean theophylline concentration in the theophylline group was 4.9 mg/l (SD 1.5) at 3‑h follow-up.

**Table 1:** Baseline patient characteristics

	Theophylline group (*N* = 13)	Control group (*N* = 11)
*Clinical*		
Mean age—years (SD)	71 (16)	67 (16)
Female sex—no. (%)	6 (46)	6 (55)
Mean NIHSS score (SD)	9 (4)	7 (3)
Hypertension—no. (%)	8 (62)	8 (73)
Diabetes mellitus—no. (%)	0 (0)	3 (27)
Hyperlipidemia—no. (%)	5 (38)	7 (64)
Arterial fibrillation—no. (%)	2 (15)	0 (0)
Peripheral arterial disease—no. (%)	1 (8)	0 (0)
Previous myocardial infarction—no. (%)	1 (8)	0 (0)
Previous transitory ischemic attack—no. (%)	2 (15)	0 (0)
Previous stroke—no. (%)	1 (8)	2 (18)
Previous intracranial hemorrhage—no. (%)	0 (0)	0 (0)
Current smoking—no. (%)	6 (46)	4 (36)
Antiplatelet agent—no. (%)	4 (31)	5 (45)
*Imaging characteristics*		
Mean volume of infarct core—ml (SD)	7.1 (8.1)	4.4 (4.5)
Mean volume of tissue at risk—ml (SD)	53.9 (65.1)	25.1 (34.7)
Large vessel occlusion—no. (%)	7 (54)	4 (36)
*Process measures*		
Mean stroke onset − door time—min (SD)	94 (53)	102 (31)
Mean door to needle-time (thrombolysis)—min (SD)	44 (12)	41 (11)
Additional endovascular therapy—no. (%)	1 (8)	2 (18)
*Follow-up characteristics*		
Mean NIHSS score at 24 h (SD)	7 (6)	5 (5)
Mean volume of final FLAIR lesion at 24 h—ml (SD)	24.8 (24.8)	14.7 (14.9)
Recanalization (TIMI 2–3) at 3 h—no. (%)^a^	3 (43)	3 (75)

The baseline characteristics and main follow-up characteristics were similar in both groups (baseline characteristics with *p*-values available in the supplemental material)

*SD* standard deviation, *NIHSS* National Institute of Health Stroke Scale, *TIMI* thrombolysis in myocardial infarction grading of arterial obstruction (score zero = complete occlusion; one = severe stenosis; two = mild to moderate stenosis; three = normal arterial caliber), *FLAIR* Fluid-attenuated inversion recovery

^a^Recanalization was achieved in 3 out of 7 patients with occlusion in the theophylline group and 3 out of 4 patients in the control group

### Infarct Core

**Fig. 2:**
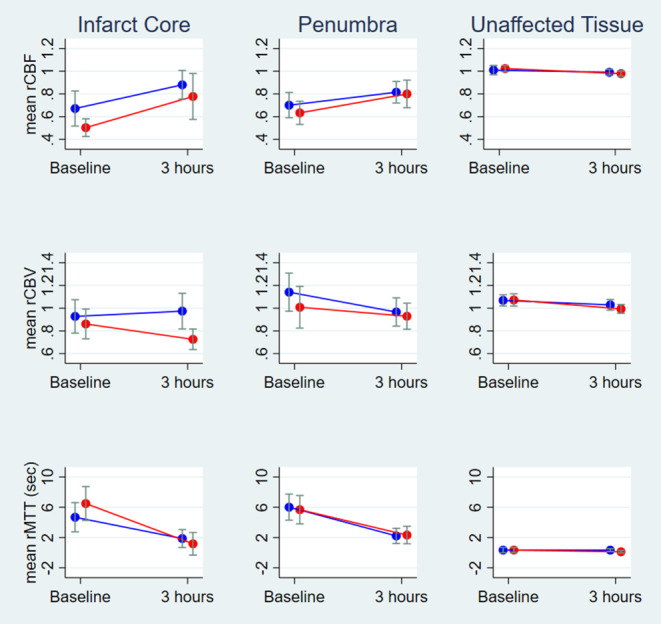
Infarct core, penumbra, and unaffected tissue with mean relative cerebral blood flow (*rCBF*), relative cerebral blood volume (*rCBV*), and relative mean transit time (*rMTT*) with confidence intervals (*blue* = theophylline group, *red* = control group) at baseline and 3‑h follow-up

The mean rCBF values in the infarct core at baseline and follow-up as well as the change from baseline to 3‑h follow-up were similar in the theophylline group and the control group (*p* = 0.47). The mean rCBV was similar at baseline (*p* = 0.34) but significantly higher at 3‑h follow-up in the theophylline group (*p* < 0.01). Likewise, there was a statistically significant difference (*p* < 0.04) regarding the mean rCBV change from baseline to 3‑h follow-up in the theophylline group (mean = 0.05; SD = 0.18) compared to the control group (mean = −0.14; SD = 0.24; Table 2). The mean ADC and rMTT values at baseline and 3‑h follow-up, and the corresponding changes from baseline to 3‑h follow-up were similar between the two groups (Fig. 2).

**Table 2:** Perfusion parameters

	Infarct core			Penumbra			Unaffected tissue		
	Theophylline (*n* = 13)	Control (*n* = 11)	*P*‑value	Theophylline (*n* = 13)	Control (*n* = 11)	*P*‑value	Theophylline (*n* = 13)	Control (*n* = 11)	*P*‑value
*Baseline*									
rCBF—(SD)	0.67 (0.28)	0.50 (0.13)	0.16^a^	0.70 (0.21)	0.63 (0.17)	0.51^a^	1.01 (0.07)	1.02 (0.04)	0.14^a^
rCBV—(SD)	0.93 (0.27)	0.86 (0.22)	0.34^a^	1.14 (0.31)	1.01 (0.31)	0.28^a^	1.06 (0.09)	1.07 (0.09)	0.75^a^
rMTT—(SD), sec	4.7 (3.6)	6.5 (3.8)	0.21^a^	6.02 (3.17)	5.68 (3.17)	0.93^a^	0.34 (0.64)	0.35 (0.60)	0.58^a^
ADC—(SD), mm^2^/s	449 (24)	450 (27)	0.66^a^	823 (179)	762 (62)	0.66^a^	826 (46)	843 (84)	0.71^a^
*3* *h follow-up*									
rCBF—(SD)	0.88 (0.23)	0.78 (0.34)	0.28^a^	0.82 (0.17)	0.80 (0.21)	0.66^a^	0.99 (0.05)	0.98 (0.04)	0.47^a^
rCBV—(SD)	0.97 (0.29)	0.73 (0.15)	0.01^a^	0.97 (0.23)	0.93 (0.38)	0.66^a^	1.03 (0.09)	0.99 (0.07)	0.34^a^
rMTT—(SD), sec	1.87 (2.17)	1.18 (2.53)	0.34^a^	2.22 (1.85)	2.34 (1.95)	0.88^a^	0.34 (0.36)	0.12 (0.29)	0.11^a^
ADC—(SD), mm^2^/s	650 (109)	610 (115)	0.34^a^	770 (72)	740 (75)	0.31^a^	798 (29)	803 (34)	0.88^a^
*Mean *∆* 0* *h–3* *h*									
rCBF—(SD)	0.21 (0.33)	0.27 (0.36)	0.65^b^	0.11 (0.21)	0.17 (0.16)	0.51^b^	−0.02 (0.05)	−0.04 (0.04)	0.20^b^
rCBV—(SD)	0.05 (0.18)	−0.14 (0.24)	0.04^b^	−0.17 (0.25)	−0.08 (0.20)	0.32^b^	−0.04 (0.07)	−0.08 (0.07)	0.18^b^
rMTT—(SD), sec	−2.82 (4.26)	−5.32 (4.43)	0.17^b^	−3.80 (4.17)	−3.35 (3.16)	0.77^b^	−0.00 (0.64)	−0.22 (0.54)	0.39^b^
ADC—(SD), mm^2^/s	201 (101)	159 (110)	0.35^a^	−53 (195)	−22 (29)	0.61^a^	−29 (54)	−40 (78)	0.67^a^

*SD* standard deviation

^a^Two-sample Wilcoxon rank-sum (Mann-Whitney) test

^b^Two-sample t test with equal variances

### Penumbra

No significant differences were found comparing the mean ADC, rCBF, rCBV, and rMTT values at baseline and 3‑h follow-up in the penumbra between the theophylline group and the control group (Table 2; Fig. 2).

### Unaffected Tissue

Likewise, the mean ADC, rCBF, rCBV, and rMTT values in the unaffected tissue at baseline and 3‑h follow-up were similar between the theophylline group and the control group (Table 2; Fig. 2).

## Discussion

Our data suggest that theophylline, as an add-on to thrombolytic therapy in acute ischemic stroke, does not significantly affect the rCBF, rCBV, or rMTT in the penumbra or the unaffected tissue. The only significant difference found between the two groups was the change in rCBV from baseline to 3‑h follow-up in the infarct core with higher values in the theophylline group and lower values in the control group. The rCBF and rMTT in the infarct core were unaffected.

The perfusion parameters CBF, CBV, and MTT are established parameters to define the infarct core and penumbra in acute ischemic stroke patients [21–23]; however, the applied thresholds to distinguish infarct core from penumbra and the prediction of outcome and final infarct volume vary across different studies [24–26]. The interpretation of the different parameters can be difficult, especially as the calculation of the perfusion parameters is complex and different methods are used in studies [23]. In ischemic lesions, uncoupling of CBV and CBF can occur in parts of the lesion with regionally reduced or increased CBV and CBF values [27]. The initial response of brain tissue to a decrease of cerebral perfusion pressure is to dilate the vessels in the ischemic area to maintain the CBF [21]. Our data showed significant changes in mean rCBV in the infarct core at 3‑h follow-up with higher values in the theophylline group. At this point, it remains speculative whether the increased rCBV in the infarct core in the theophylline group is due to the positive chronotropic and inotropic effect of theophylline; however, this theory might be supported by our finding of a significant difference in heart rate with higher values in the theophylline group, which could be an indicator for increased cardiac output with subsequent increased mean arterial pressure, increased cerebral perfusion pressure, and finally increased rCBV. Another explanation might be the inverse intracerebral steal (the so-called Robin Hood phenomenon) with vasoconstriction and decreased CBF in healthy brain tissue and increased CBF in ischemic tissue previously described by Skinhøj and Paulson, which might also have an effect of CBV [11, 12]; however, our results do not really support this explanation, as no significant rCBF change in the infarct core, and especially no decrease of rCBF in the unaffected tissue, was observed from baseline to 3‑h follow-up. Even more important, the rCBF, rCBV, and rMTT in the penumbra was not significantly different between the theophylline group and control group. The lack of improved perfusion in the salvageable penumbra might explain absence of a clinical effect and failure to reduce the final infarct volume by theophylline [16] as the infarct core was the only region which showed significant differences in rCBV but is commonly assumed to be non-salvageable. It should also be mentioned that there is experimental evidence that adenosine released by astrocytes during ischemia might have a protective effect on neurons [28] and microcirculation [9]. Thus, the inhibition of the adenosine receptor by theophylline might have a counterproductive effect.

A comparison of our data with previous studies is difficult, as patient selection, trial medication application, and imaging techniques are substantially different. These studies demonstrated intracerebral vasoconstriction and decreased CBF when theophylline was applied as bolus injection into the internal carotid artery [11] or intravenously [12], whereas a short intravenous infusion of theophylline over 15 min was used in our study. The previous studies measured the CBF almost immediately after administration of theophylline in contrast to our measurements after 3–4 h; however, the average serum theophylline concentration of 4.9 mg/l in our trial is comparable with the concentration found in the study of Magnussen et al. [29]. The authors reported a serum theophylline concentration of about 5 mg/l and a 22% reduced global CBF 45 min after bolus injection of 250 mg of theophylline. Thus, it remains uncertain if the different types of theophylline administrations and/or the timing of the CBF measurements can explain the controversial results.

The strength of our study compared to previous studies is a dataset from a randomized, placebo-controlled trial design with MRI-verified ischemic stroke patients. The main limitation of our analysis is the small number of patients included in this secondary study caused by the early termination of the main trial due to slow recruitment. Thus, the varying infarct core and penumbra volumes, with and without large vessel occlusion, and with and without revascularization at follow-up is a limitation. Another limitation might be our definition of the infarct core and the penumbra. Spreading depolarization, often a single terminal event that mediates neuronal death in the infarct core, causes cytotoxic edema with restricted diffusion in the intracellular and extracellular spaces that decrease the signal in the ADC maps [30]; however, a normalization of the ADC lesion can be seen in cases of tissue reperfusion, especially within 3 h after stroke onset [31]. Thus, due to the dynamics of the ADC lesion, the infarct core definition in this study might limit the interpretability of the perfusion changes in the infarct core; however, our data did not show perfusion changes in the penumbra and unaffected brain tissue, which contradicts the hypothesis of the inverse intracerebral steal, namely vasoconstriction of the healthy tissue in favor for the salvageable penumbra (Robin Hood phenomenon). These findings might explain the lack of clinical improvement after 3‑h follow-up in this study, lack of early clinical improvement or reduced infarct growth at 24‑h follow-up in the main trial [16] and lack of clinical improvement in previous clinical trials [4, 5].

In conclusion, our results indicate that theophylline does not change the perfusion in potentially salvageable penumbra in acute ischemic stroke, but only affects the rCBV in the infarct core. In contrast to penumbral tissue, the infarct core is unlikely to be salvageable, which might explain why theophylline fails to reduce the final infarct volume and fails to improve the clinical outcome.

## Supplementary Information

Table 1 Baseline patient characteristics
