# Supplementary material for: Perfusion Changes in Acute Stroke Treated with Theophylline as an Add-on to Thrombolysis: A Randomized Clinical Trial Subgroup Analysis
Source: Clin Neuroradiol. 2021 Jul 14;32(2):345–52. doi: 10.1007/s00062-021-01029-x (PMC9187573; doi:10.1007/s00062-021-01029-x)
Supplement: Supplementary file 1 — Table 1 Baseline patient characteristics [file 62_2021_1029_MOESM1_ESM.docx]

**Supplemental material**

| Table 1. Baseline Patient Characteristics | Theophylline group  (N =13) | Control group  (N =11) | P value |
| --- | --- | --- | --- |
| *Clinical* |  |  |  |
| Mean age— year (SD) | 71 (16) | 67 (16) | 0.26^a^ |
| Female sex — no. (%) | 6 (46) | 6 (55) | 1^b^ |
| Mean NIHSS score (SD) | 9 (4) | 7 (3) | 0.35^c^ |
| Hypertension — no. (%) | 8 (62) | 8 (73) | 0.68^b^ |
| Diabetes mellitus — no. (%) | 0 (0) | 3 (27) | 0.08^b^ |
| Hyperlipidemia — no. (%) | 5 (38) | 7 (64) | 0.41^b^ |
| Arterial fibrillation — no. (%) | 2 (15) | 0 (0) | 0.48^b^ |
| Peripheral arterial disease — no. (%) | 1 (8) | 0 (0) | 1^b^ |
| Previous myocardial infarction — no. (%) | 1 (8) | 0 (0) | 1^b^ |
| Previous transitory ischemic attack — no. (%) | 2 (15) | 0 (0) | 0.48^b^ |
| Previous stroke — no. (%) | 1 (8) | 2 (18) | 0.58^b^ |
| Previous intracranial hemorrhage — no. (%) | 0 (0) | 0 (0) | 1^b^ |
| Current smoking — no. (%) | 6 (46) | 4 (36) | 0.70^b^ |
| Antiplatelet agent — no. (%) | 4 (31) | 5 (45) | 0.70^b^ |
| *Imaging characteristics* |  |  |  |
| Mean volume of infarct core— ml (SD) | 7.1 (8.1) | 4.4 (4.5) | 0.43^c^ |
| Mean volume of tissue at risk – ml (SD) | 53.9 (65.1) | 25.1 (34.7) | 0.26^c^ |
| Large vessel occlusion— no. (%) | 7 (54) | 4 (36) | 0.44^b^ |
| *Process measures* |  |  |  |
| Mean stroke onset – door time — min (SD) | 94 (53) | 102 (31) | 0.40^c^ |
| Mean door to needle-time (thrombolysis) — min (SD) | 44 (12) | 41 (11) | 0.45^c^ |
| Additional endovascular therapy — no. (%) | 1 (8) | 2 (18) | 0.58^b^ |
| *Follow-up characteristics* |  |  |  |
| Mean NIHSS score at 24h (SD) | 7 (6) | 5 (5) | 0.98^d^ |
| Mean volume of final FLAIR-lesion at 24h— ml (SD) | 24.8 (24.8) | 14.7 (14.9) | 0.51^c^ |
| Recanalization (TIMI 2-3) at 3h — no. (%) * | 3 (43) | 3 (75) | 0.54^b^ |
| Death within 90 days — no. (%) | 0 (0) | 0 (0) |  |

The baseline characteristics and main follow-up characteristics were similar in both groups

a= Two-sample t test with equal variances, b= Fisher´s exact test, c= Two-sample Wilcoxon rank-sum (Mann-Whitney) test, d= adjusted for stratification factors age and stroke severity and predefined adjustment factors,

* Recanalization was achieved in 3 out of 7 patients with occlusion in the theophylline group and 3 out of 4 patients in the control group

Abbreviations: SD = standard deviation, NIHSS = National Institute of Health Stroke Scale, TIMI = thrombolysis in myocardial infarction grading of arterial obstruction (score zero=complete occlusion; one=severe stenosis; two=mild to moderate stenosis; three=normal arterial caliber)
